# Mechanisms of Action of the KCa2-Negative Modulator AP30663, a Novel Compound in Development for Treatment of Atrial Fibrillation in Man

**DOI:** 10.3389/fphar.2020.00610

**Published:** 2020-05-06

**Authors:** Bo Hjorth Bentzen, Sofia Hammami Bomholtz, Rafel Simó-Vicens, Lasse Folkersen, Lea Abildgaard, Tobias Speerschneider, Kalai Mangai Muthukumarasamy, Nils Edvardsson, Ulrik S. Sørensen, Morten Grunnet, Jonas Goldin Diness

**Affiliations:** ^1^Acesion Pharma, Copenhagen, Denmark; ^2^Department of Biomedical Sciences, Faculty of Health and Medical Sciences, University of Copenhagen, Copenhagen, Denmark; ^3^Institute of Biological Psychiatry, Sankt Hans Hospital, Roskilde, Denmark; ^4^Department of Molecular and Clinical Medicine/Cardiology, Institute of Medicine, Sahlgrenska Academy, University of Gothenburg, Gothenburg, Sweden

**Keywords:** atrial fibrillation, ion channels, anti-arrhythmic drugs, SK channels, K_Ca_2

## Abstract

**Aims:**

Small conductance Ca^2+^-activated K^+^ channels (SK channels, K_Ca_2) are a new target for treatment of atrial fibrillation (AF). AP30663 is a small molecule inhibitor of K_Ca_2 channels that is currently in clinical development for treatment of AF. The aim of this study is to present the electrophysiological profile and mechanism of action of AP30663 and its efficacy in prolonging atrial refractoriness in rodents, and by bioinformatic analysis investigate if genetic variants in *KCNN2* or *KCNN3* influence the expression level of these in human heart tissue.

**Methods and Results:**

Whole-cell and inside-out patch-clamp recordings of heterologously expressed K_Ca_2 channels revealed that AP30663 inhibits K_Ca_2 channels with minor effects on other relevant cardiac ion channels. AP30663 modulates the K_Ca_2.3 channel by right-shifting the Ca^2+^-activation curve. In isolated guinea pig hearts AP30663 significantly prolonged the atrial effective refractory period (AERP) with minor effects on the QT-interval corrected for heart rate. Similarly, in anaesthetized rats 5 and 10 mg/kg of AP30663 changed the AERP to 130.7±5.4% and 189.9±18.6 of baseline values. The expression quantitative trait loci analyses revealed that the genome wide association studies for AF SNP rs13376333 in *KCNN3* is associated with increased mRNA expression of *KCNN3* in human atrial appendage tissue.

**Conclusions:**

AP30663 is a novel negative allosteric modulator of K_Ca_2 channels that concentration-dependently prolonged rodent atrial refractoriness with minor effects on the QT-interval. Moreover, AF associated SNPs in *KCNN3* influence *KCNN3* mRNA expression in human atrial tissue. These properties support continued development of AP30663 for treatment of AF in man.

## Introduction

Atrial fibrillation (AF) is the most common cardiac arrhythmia affecting more than 30 million people worldwide, a number that is rising partly because of the aging population and better detection. AF is associated with 2- and 1.5 fold increases in risk of all-cause mortality in woman and men respectively, and an increased risk of heart failure and stroke (Kirchhof et al., 2016). Management of patients with AF has improved, but pharmacological rhythm control therapy is still limited by moderately effective drugs with potentially serious extra-cardiac or ventricular adverse effects (Waks and Zimetbaum, 2016). Hence, novel pharmacological rhythm control therapies are warranted.

The small conductance calcium activated potassium channel (SK, K_Ca_2), encoded by the *KCNNX* gene is a new drug target for treatment of AF (Heijman and Dobrev, 2017). As the name implies, K_Ca_2 channels are potassium channels activated by intracellular calcium. Three subtypes of K_Ca_2 channel alfa-subunits exists (K_Ca_2.1–3, SK1–3) and in the human atria K_Ca_2.2 and K_Ca_2.3 predominate (Skibsbye et al., 2014). Genome wide association studies (GWAS) for AF have identified single nucleotide polymorphisms (SNPs) in *KCNN2* and *KCNN3* that are highly associated with AF (Ellinor et al., 2010; Ellinor et al., 2012; Christophersen et al., 2017). Preclinical studies showed that K_Ca_2 channels during sinus rhythm as well as during AF play a more prominent role in atria as compared to ventricles in several species including man (Tuteja et al., 2005; Li et al., 2009; Diness et al., 2010; Qi et al., 2014; Skibsbye et al., 2014; Haugaard et al., 2015; Diness et al., 2017), thereby exhibiting a functional atrial specificity. In atrially tachy-paced pigs that were resistant to pharmacological cardioversion of AF with vernakalant, negative allosteric modulation of the K_Ca_2 channel converted AF to sinus rhythm (Diness et al., 2017). In the current study, we first investigate if genetic variants (SNPs) in *KCNN2* or *KCNN3* found in GWAS to be associated with AF influence the expression level of *KCNN2* or *KCNN3* in human atrial or ventricular tissue. Next, we present the ion channel profile, mode of action, and *in vitro*, *ex vivo* and *in vivo* effects of the clinical candidate AP30663.

## Materials and Methods

### Expression Quantitative Trait Loci Analyses

The expression quantitative trait loci analyses (eQTL) effects of the AF GWAS associated SNP rs337711 on *KCNN2* and SNP rs13376333 on *KCNN3* were investigated in the Genotype-Tissue Expression (GTEx) database, release v05-08-15, using default dashboard analytics setup. GTEx data gene expression was measured using RNA-sequencing with Illumina TruSeq library and genotyping was done using whole genome sequencing on an illumina HiSeq X machine (Carithers et al., 2015). For eQTL replication we used the Advanced Study of Aortic Pathology (ASAP) cohort of left-ventricular heart tissue, measured using Affymetrix ST 1.0 Arrays and genotyping was done using Illumina Human 610W-Quad Beadarrays (Folkersen et al., 2011). The statistical calculation was performed using a linear additive model, where genotype was encoded as 0, 1, or 2 and used as explanatory variable, with gene expression level as response variable.

### *In Vitro* Electrophysiology

The potency of AP30663 was assessed in HEK cells stably expressing rat Na_V_1.5 or human Ca_V_1.2, K_Ca_2.1, K_Ca_2.2 or K_Ca_2.3 channels and CHO cell line stably expressing human K_V_11.1 using the automated patch-clamp system, Qpatch 16 (Sophion, Ballerup, Denmark) at room temperature. The effect of AP30663 on heteromeric K_V_11.1a and K_V_11.1b channels was addressed by manual patch clamping. Inside out patch-clamp recordings were performed on HEK cells stably expressing human K_Ca_2.3 channels using a HEKA EPC9 amplifier and the Patchmaster software (HEKA Elektronik, Germany). Effects of AP30663 on late Na_V_1.5 currents were addressed by manual whole-cell patch-clamping using HEK cells transiently transfected with human Na_V_1.5 in the absence and presence of ATXII. The effect of AP30663 was also assessed on the currents conducted by Kir2.1 (I_K1_); K_V_7.1/KCNE1 (I_ks_); K_V_4.3/KCHIP2 (I_to_), K_ir_3.1/K_ir_3.4 (I_KACh_); and K_V_1.5 (I_Kur_) using the two-electrode voltage-clamp method on Xenopus oocytes, as previously described (Diness et al.). See details of *in vitro* electrophysiology methods in **Supplementary Methods and Materials**.

### Animal Experiments

Animal experiments were performed under the license from the Danish Ministry of justice (2018-15-0201-01430 & 2016-15-0201-00850) and in accordance with the Danish guidelines for animal experiments according to the European Commission Directive 86/609/EEC.

#### Isolated Perfused Guinea Pig Heart Experiments

A total of 12 female and 6 male Guinea pig hearts were used. In brief after anesthesia the hearts were removed and retrogradely perfused with Krebs–Henseleit solution via the aorta. Hearts were mounted in a Langendorff set-up (Hugo Sachs Elektronik, Germany) and electrocardiograms (ECG) were obtained with three ECG electrodes placed near the heart. A pacing electrode on the right atrium was used to stimulate the atria and measure atrial refractory periods (AERP).

After the baseline recording, three 20-min episodes followed in which the heart was perfused with increasing concentrations of AP30663 (1, 3, and 10 µM). Time matched control hearts underwent the same procedure. For details see **Supplementary Methods**.

#### Irwin

An observational Irwin test for assessing CNS exposure of AP30663 was performed in mice. A total of three NMRI mice (Taconic Europe, Lille Skensved, Denmark) weighing 40 to 60 g were used. After injection of a bolus dose of 10 mg/kg AP30663 in the tail vein, the mice were observed for up to 30 min.

#### Closed Chest Rat AERP

A total of seven male Sprague–Dawley rats were used for the closed chest preparations. The rats were anaesthetized with 3 % isofluran/oxygen and catheterized to allow for drug infusion and intra cardiac pacing. The AERP was measured by applying electrical stimulation. Baseline AERP recordings were made every fifth minute for 20 min. Hereafter, two 20-min episodes followed in which the animal was injected with increasing doses of AP30663 (5 and 10 mg/kg) or equivalent volumes of vehicle. AERP was measured 0.5, 2, 4, 6, 10, and 15 min after each injection. For details see **Supplementary Methods**.

### Drugs and Solutions

AP30663 was solubilized in DMSO stock concentrations of 10 mM for *in vitro* experiments. The highest concentration of DMSO was 0.1%. For *in vivo* experiments, 5 mg/ml AP30663 were dissolved in a vehicle consisting of 50% polyethylene glycol (PEG) 400 (Merck, Germany) and 50% sterile saline (PanReac AppliChem, Germany).

### Data Analysis

*In vitro* data were extracted from PatchMaster or Sophion QPatch Assay Software and analyzed using GraphPad Prism 7. For detailed description, see **Supplementary Methods**. Data are summarized as mean ± SEM.

*In vivo* and *ex vivo* experiments were analyzed using GraphPad Prism software. LabChart software was used for analyzing the following ECG parameters: QT, PR, RR, QRS. The QT-interval corrected for heart rate, QTcH, was calculated as described in Holzgrefe et al. (2014) using the guinea pig specific formula:

$$\text{QTcH}=\mathrm{QT}/(\mathrm{RR}/0.28{)}^{0.7861}.$$

Continuous data are summarized using the mean ± SEM. Multiple unpaired t-tests without assuming consistent standard deviations and using Holm-Sidaks correction for multiple comparisons were used to compare the AP30663 groups to their respective TMC. *P* values < 0.05 were considered significant and are given with three decimals. Manuscript Formatting.

## Results

### AF-Associated SNPs in KCNN3 Influences KCNN3 mRNA Expression in Human Atrial Tissue

**Figure 1:**
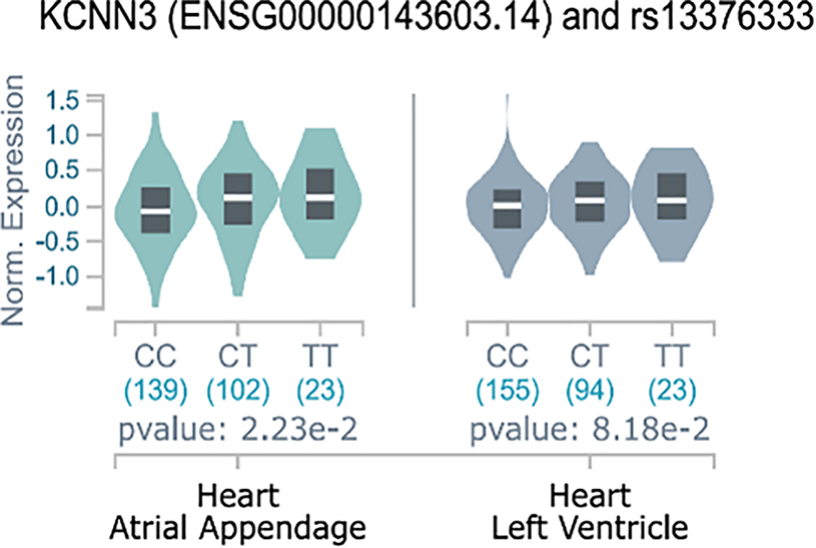
eQTL effects of rs13376333 on KCNN3. Y-axis indicates normalized expression value of *KCNN3* according to the GTEx standard analysis pipeline (Carithers et al., 2015). The figure shows that the expression of KCNN3 in atrial appendage is increased in the 23 GTEx individuals with the rs13376333-T/T genotype, as compared to both the 102 heterozygote (C/T) GTEx individuals and the 139 individuals that are major allele homozygote (C/C). Having the T-allele have also been shown to increase risk of AF (Ellinor et al., 2010; Ellinor et al., 2012; Christophersen et al., 2017). These findings were replicated in an independent eQTL data (*P* = 0.0162, n = 127).

We found that the minor allele T of SNP rs13376333 was associated with higher mRNA expression of *KCNN3* in the GTEx atrial appendage samples (**Figure 1**) (*P* = 0.0223, n = 264). In the one other GTEx heart-related tissue available, the left ventricle, there was also a trend towards increased expression with the minor allele A, although not statistically significant (*P* = 0.0818, *n* = 272). Additionally, we observed the same effect in the lung tissue (*P* = 2.13e-8) and in one brain region tissue (the BA24 region, P=0.0162). The cardiac eQTL effect was replicated in independent samples of left ventricular biopsies from patients undergoing surgery in the ASAP study, also with the minor allele T resulting in increased expression (P=0.0162, n= 127). In comparison SNP rs337711 was not associated with atrial or ventricular changes in *KCCN2* mRNA expression (*P* = 0.6 and *P* = 0.1, respectively).

### AP30663 Inhibits K_Ca_2 Channels by Modifying the Calcium Sensitivity of the Channel

**Figure 2:**
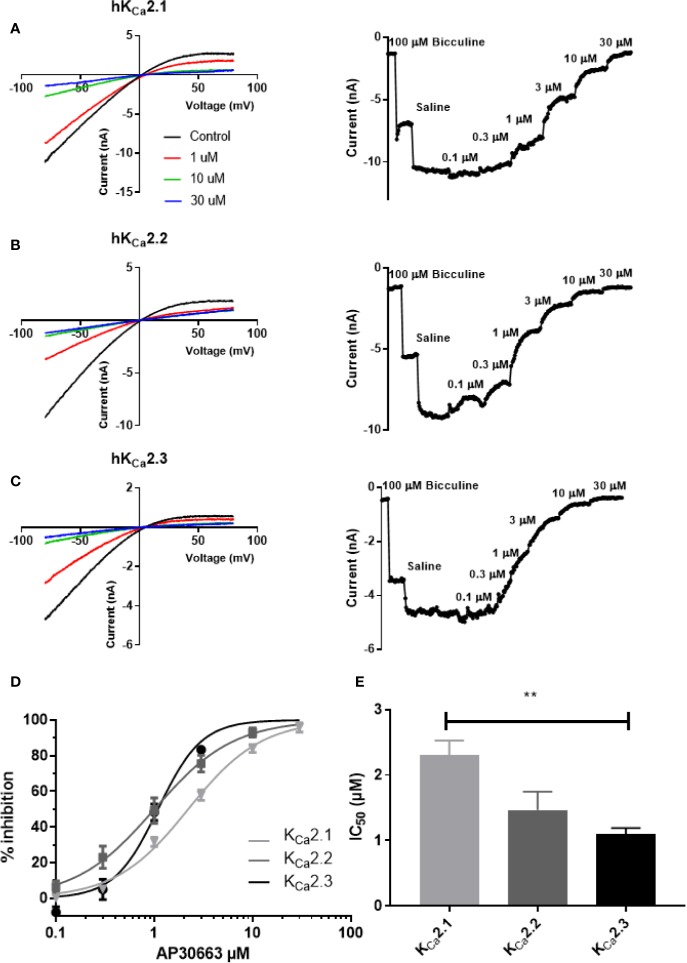
AP30663 inhibits hK_Ca_2 channels. The effect of increasing concentrations of AP30663 was investigated by automated whole-cell patch-clamping in HEK cells stably expressing human K_Ca_2 channels. Currents were elicited by applying a depolarizing voltage ramp protocol from −80 mV to +80 mV for 200 ms from a holding potential of 0 mV in symmetrical K^+^ solutions. Current–voltage recordings are depicted in the left panel for K_Ca_2.1 **(A)**, K_Ca_2.2 **(B)** and K_Ca_2.3 **(C)** before (black) and after application of 1 µM (red), 10 µM (green) and 30 µM (blue) AP30663. Current-time plots (right panel) showing the effect of increasing concentrations of AP30663 on the for K_Ca_2.1 (A), K_Ca_2.2 **(B)**, and K_Ca_2.3 **(C)** currents measured at a membrane potential of −80 mV. **(D)** Concentration–response curve of AP30663 on K_Ca_2.1 (light gray), K_Ca_2.2 (dark gray), and K_Ca_2.3 (black). **(E)** Comparison of IC_50_ values for the 3 K_Ca_2 channel subtypes. (K_Ca_2.1, n = 9; K_Ca_2.2, n = 19; and K_Ca_2.3, n = 16). **p < 0.01.

AP30663 inhibited the K_Ca_2 current in a concentration-dependent manner (**Figures 2A–C**). The concentration response curves show that AP30663 inhibits all K_Ca_2 channel subtypes but with a slightly lower potency on K_Ca_2.1 (IC_50_= 2.29 ± 0.22 µM; K_Ca_2.2, IC_50_ =1.46 ± 0.28 µM; K_Ca_2.3, IC_50_= 1.09 ± 0.09 µM) (see **Figures 2D, E**).

**Figure 3:**
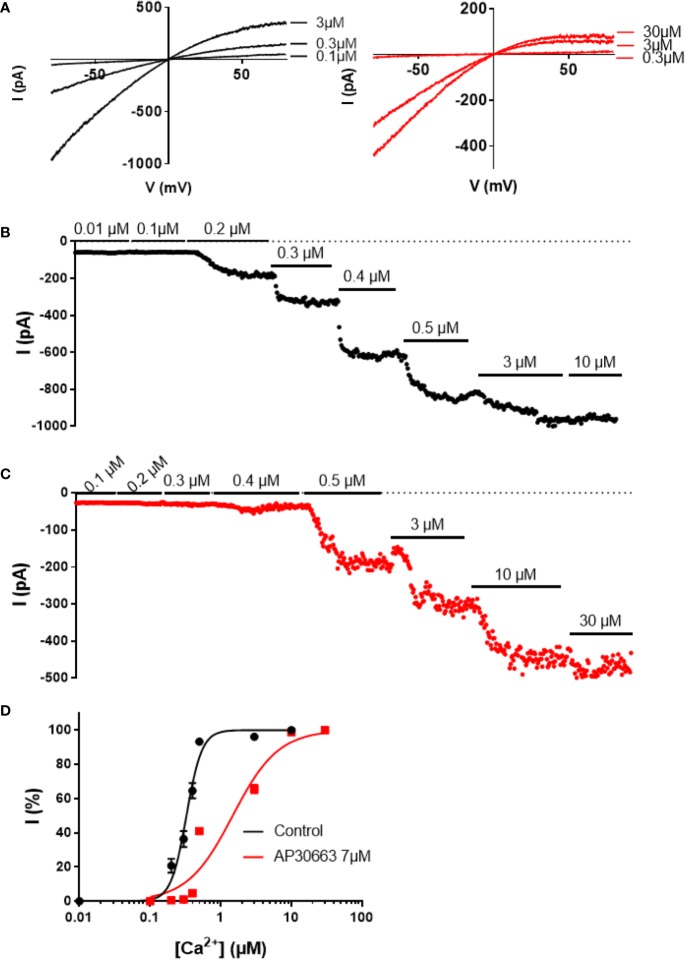
AP30663 shifts the calcium activation curve of hK_Ca_2.3 channels. Current-voltage recordings **(A)** and corresponding current-time plots of K_Ca_2.3 measured using the inside-out patch-clamp exposed to different free calcium concentrations, in the absence **(B)** (black) or presence of AP30663 7 µM **(C)** (red). **(D)** Calcium activation curves for K_Ca_2.3 in the absence (black, n = 6) or presence of AP30663 7 µM (red, n = 6). Currents were measured at a membrane potential of −80 mV.

To investigate the inhibitory mechanism of AP30663, we performed inside out patch-clamp experiments on HEK cells expressing K_Ca_2.3 (**Figure 3**). In the absence of AP30663 the K_Ca_2.3 channel had an EC_50_ for calcium activation of 0.33 ± 0.02 µM and was fully active at 3 µM. However, in the presence of 7 µM AP30663, 10 µM of calcium is needed to achieve full activation, and the EC_50_ for calcium activation is shifted to 1.50 ± 0.12 µM (EC_50_ baseline vs. AP30663: *P* < 0.0001). In addition to shifting the calcium activation curve, meaning that higher concentrations of calcium are needed to activate the channel, AP30663 also significantly decreased the Hill slope of the calcium activation curve from 4.4 ± 0.5 to 1.4 ± 0.1 (slope baseline vs. AP30663: *P* < 0.0001).

### Ion Channel Selectivity Profile of AP30663

**Figure 4:**
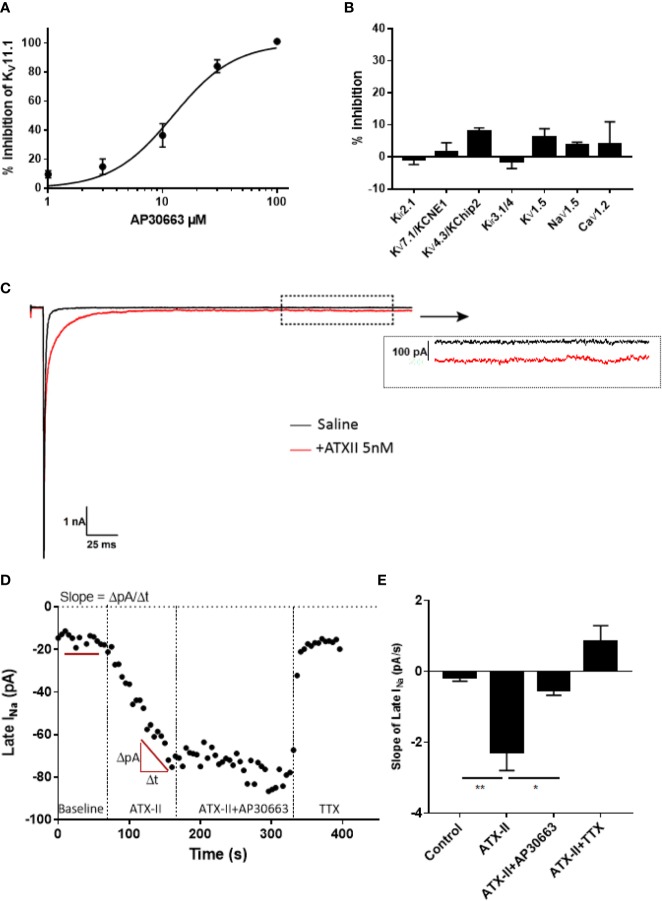
Ion channel selectivity profile of AP30663. **(A)** Concentration-response curve of the effect of increasing concentrations of AP30663 on K_V_11.1a recorded by automated whole-cell patch-clamp (n = 4). **(B)** % inhibition by 10 µM AP30663 on K_ir_3.1/K_ir_3.4 (I_KACh_, n = 6), K_V_1.5 (I_Kur_, n = 5), K_V_7.1/KCNE1 (I_Ks_, n = 5), K_V_4.3/KChiP2 (I_to_, n = 5), and K_ir_2.1 (I_K1_, n = 5) using two-electrode voltage-clamp and Na_V_1.5 (I_Na_, n = 11), Ca_V_1.2 (I_Ca_, n = 3; 30 µM) using automated whole-cell patch-clamping. **(C)** Current traces of the whole-cell Na^+^ current before and after application of ATXII recorded from HEK293 cells transiently transfected with hNa_V_1.5 (dotted box demonstrates where the late-sodium current was analyzed). **(D)** Late-sodium current amplitudes as a function of time. **(E)** Effect of compounds on the slope of the late-sodium current vs time plot. The slope was found by linear regression on the last 10 points of each liquid period (n = 14).

AP30663 was tested on a panel of cardiac ion channels to determine the functional selectivity profile of the compound. Automated whole-cell patch-clamp recordings of hK_V_11.1a channels revealed that AP30663 inhibited the I_Kr_ current with an IC_50_ value of 15.1 ± 2.1 µM (**Figure 4A**). In comparison when tested by manual patch clamp at 35°C, on HEK cells transfected with both cardiac isoforms K_V_11.1a and K_V_11.1b, AP30663 was estimated to have an IC_50_ of 4.0 ± 1.5 µM. 10 µM AP30663 did not significantly affect K_ir_3.1/K_ir_3.4 (I_KACh_), K_V_1.5 (I_Kur_), K_V_7.1/KCNE1 (I_Ks_), K_V_4.3/KChiP2 (I_to_) and K_ir_2.1 (I_K1_) channels **Figure 4B**). AP30663 did not significantly inhibit the current mediated by Ca_V_1.2 (I_Ca_) (inhibition by 30 µM: 4±7 %). The effects on peak Na_V_1.5 channel current (I_Na_) were investigated by automated patch-clamp experiments, and AP30663 in 10 µM did not affect I_Na_ (4±1% inhibition) (**Figure 4B**).

To determine if AP30663 had any effect on the late I_Na_, we conducted a set of manual patch-clamp experiments on HEK cells transfected with the human Na_V_1.5 channel. Because of its small size, it is customary to co-apply the sea anemone toxin ATXII to augment late I_Na_, and to use the sodium channel toxin tetrodotoxin (TTX) at the end of the experiment to inhibit residual late I_Na_ (Isenberg and Ravens, 1984; Maltsev et al., 1998). In **Figure 4C**, it can be seen how ATXII slows the inactivation of Na_V_1.5 and causes a small but relevant increase of the late current. From the time plot in **Figure 4D**, it can be appreciated that ATXII in our hands causes a continuous increase in the late I_Na_ amplitude. Hence, a steady state current is never achieved, and therefore judging the effect of AP30663 solely based on changes in late I_Na_ amplitude would be difficult. To circumvent this, we quantified the effect of 10 µM AP30663 on ATXII augmented late I_Na_ by comparing the changes in the slope of the late I_Na_ vs time plot. From the bar graph that summarizes the effect of AP30663 on the slope from 14 experiments it can be concluded that 10 µM AP30663 inhibits the late I_Na_ (**Figure 4E**).

### AP30663 Prolongs the Atrial Refractory Period With Small Effects on Ventricular Repolarization—Functional Atrial Selectivity

**Figure 5:**
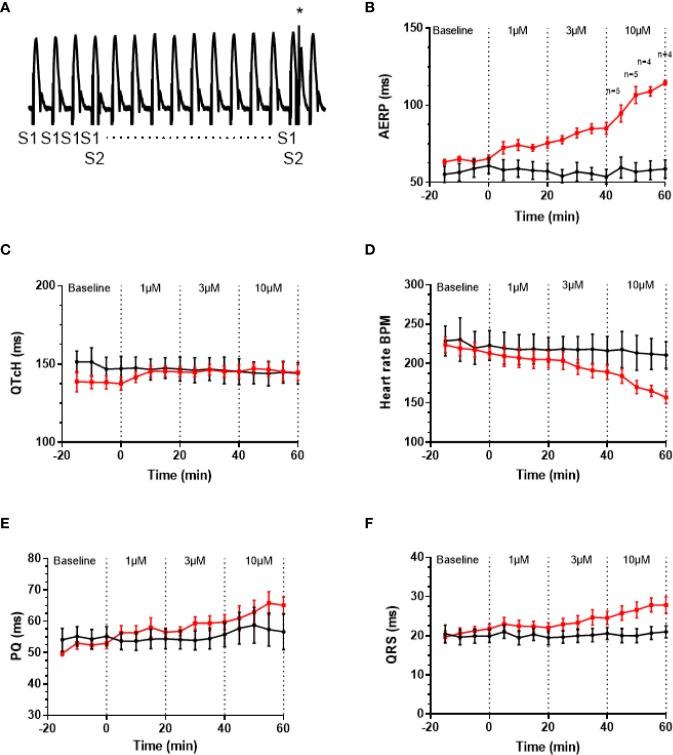
AP30663 prolongs the atrial refractory period in isolated perfused female guinea pig hearts. **(A)** Monophasic action potential recording recorded from the atrium of an isolated perfused guinea pig heart, during investigation of the atrial effective refractory period (AERP). Ten pacing stimuli (S1) are delivered at fixed basic cycle length of 200 ms, and for every 10th beat, an extra stimulus (S2 marked by * in the recording) is applied with 1 ms increments (for clarity not all S2 are shown). Notice how the first S2 does not result in an atrial action potential, whereas the second S2 (+1 ms) elicits an action potential. Graph demonstrating the effect of increasing concentrations of AP30663 on AERP **(B)**, QTcH-interval **(C)**, heart rate **(D)**, PQ-interval **(E)**, and QRS-duration **(F)** as compared with time matched control experiments (TMC) (AP30663, n = 6; TMC, n = 6).

A well-known anti-arrhythmic mechanism of rhythm control therapeutics is to prolong atrial refractoriness. After a baseline period the effects of increasing concentrations of AP30663 (1, 3, and 10 µM) on AERP in isolated female guinea pig hearts were investigated. From **Figure 5**, it can be appreciated that AP30663 increases AERP in a concentration-dependent manner, while QTcH is prolonged to a much lesser degree. To take into account baseline differences we evaluated the effect of AP30663 by comparing Δdrug-baseline between TMC and AP30663 groups for each concentration (**Table 1**). From this it can be observed that AP30663 significantly prolongs AERP at all concentrations, slows the heart rate in 3 and 10 µM, prolongs the QRS in 10 µM and prolongs the PQ-interval and QTcH-interval. The latter albeit to a much lesser extent (~8 ms) as compared to the effects of AP30663 on atrial refractoriness (10–50 ms). Similar experiments were conducted in male guinea pigs. No sex differences were observed (see **Supplementary Figure 1** and **Supplementary Table 1**).

**Table 1:** Effect of AP30663 on isolated perfused female guinea pig hearts.

AP30663 concentration		1µM	3µM	10µM
AERP (ms)	TMC	−4 ± 1	−7 ± 1	−2 ± 2
	AP30663	10 ± 3	20 ± 3	47 ± 4
	*P*	0.002	<0.001	<0.001
QTcH (ms)	TMC	0 ± 1	−2 ± 2	−3 ± 3
	AP30663	8 ± 2	8 ± 3	7 ± 5
	*P*	0.029	0.053	0.099
HR (BPM)	TMC	−6 ± 3	−7 ± 4	−12 ± 5
	AP30663	−8 ± 4	−24 ± 4	−56 ± 2
	*P*	0.672	0.019	<0.001
QRS (ms)	TMC	0 ± 1	1 ± 1	1 ± 1
	AP30663	0 ± 0	3 ± 1	6 ± 1
	*P*	0.392	0.203	0.007
PQ (ms)	TMC	−1 ± 1	1 ± 1	1 ± 3
	AP30663	4 ± 2	7 ± 2	12 ± 2
	*P*	0.035	0.024	0.024

Changes in AERP, QTcH, HR, PQ, and QRS (Δdrug, baseline) for each group (TMC and AP30663) calculated at the end of drug period (20, 40, and 60 min). P values refer to the comparison of Δ values between TMC and AP30663 groups.

### AP30663 Prolongs Atrial Refractoriness *In Vivo*

**Figure 6:**
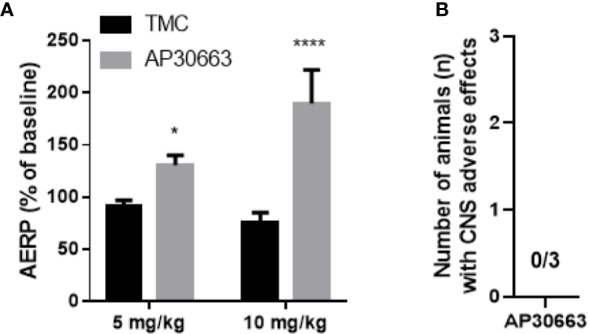
AP30663 prolongs the atrial refractory period *in vivo* in rats and does not induce CNS related adverse effects in mice. **(A)** The effect of i.v. injection of AP30663 or vehicle (time matched control, TMC) on AERP evaluated in anaesthetized rats using an intracardiac pacing catheter positioned in the right atria (TMC, n = 4; AP30663, n = 3). **(B)** Bar graph depicting the number of mice with CNS related adverse effects triggered by the i.v administration of AP30663 (10 mg/kg), n = 3. *0,0187, ****< 0,0001.

To investigate the *in vivo* effects of AP30663, male rats were infused with increasing doses of AP30663 (5 and 10 mg/kg) or corresponding volumes of vehicle (1 and 2 ml/kg), while intracardiac recordings of AERP were performed. The AERP was significantly prolonged by 5 mg/kg and 10 mg/kg of AP30663 to 143% and 250% of TMC values (**Figure 6**).

### AP30663 Does Not Impair Coordination in Mice

K_Ca_2 channels are expressed in the brain including cerebellum, and inhibition of these may lead to motor function impairment (Simó-Vicens et al., 2017). An observational Irwin test for assessing CNS exposure of AP30663 was performed in mice. Neither CNS related adverse effects nor other adverse effects were observed within 30 min of injection of 10 mg/kg i.v. AP30663 (*n* = 3, *data not shown*).

## Discussion

K_Ca_2 channels are widely expressed in the body. In the heart, K_Ca_2 channels are functionally more important for repolarization of the atria as compared to the ventricles (Diness et al., 2010; Qi et al., 2014; Skibsbye et al., 2014; Zhang et al., 2014; Haugaard et al., 2015; Diness et al., 2017). Pharmacological experiments on mice, rats, guinea pigs, rabbits, pigs, goats, dogs and horses have demonstrated that K_Ca_2 channel inhibitors can prolong atrial refractoriness, terminate AF and prevent reinduction of AF (Diness et al., 2010; Diness et al., 2011; Qi et al., 2014; Haugaard et al., 2015; Gatta et al., 2019). In addition, we previously demonstrated that K_Ca_2 channel inhibition can cardiovert vernakalant-resistant AF in atrially tachypaced pigs (Diness et al.), and prolong atrial but not ventricular APD in human multicellular preparations (Skibsbye et al., 2014). Moreover, genome wide association studies have put the K_Ca_2 channel (*KCNN2* and *KCNN3*) on the list of genes that are highly associated with AF (Ellinor et al., 2010; Ellinor et al., 2012; Christophersen et al., 2017). K_Ca_2 channels thus constitute an interesting novel drug target for treatment of AF.

The advances of genomics and transcriptomics allow us to study possible associations between genetic variants and their influence on mRNA expression levels (eQTL analyses), and hence guide drug discovery. Here we performed eQTL analyses and found that the AF-increasing variant of rs13376333 is associated with increased mRNA expression of *KCNN3* in human atrial tissue. This suggests that increased K_Ca_2 channel mRNA is associated with AF, and hence points towards the possibility of developing K_Ca_2 channel inhibitors for the treatment of AF. To this end, we have developed AP30663. AP30663 is an inhibitor of K_Ca_2 channels with no K_Ca_2-subtype selectivity amongst K_Ca_2.2 and K_Ca_2.3 but a slightly lower potency on K_Ca_2.1, which is the K_Ca_2 subtype found to be least expressed in the human heart (Skibsbye et al., 2014). AP30663 inhibits the K_Ca_2 channels when applied to both the intra- and extracellular side of the plasma membrane. From inside-out recordings we found that AP30663 significantly shifts the EC_50_ for calcium activation and lowers the Hill-slope, suggesting that AP30663 decreases the calcium sensitivity of K_Ca_2 channels thereby acting as a negative allosteric modulator of the channel. This is similar to what has been reported for two other K_Ca_2 inhibitors, NS8593 and AP14145 (Strøbaek et al., 2006; Simó-Vicens et al., 2017, 14145). Likewise, a changed Hill-slope was also observed for NS8593 and AP14145 and may indicate a loss of calcium cooperativity. AP30663 was functionally tested on a broad panel of other cardiac ion channels, and 10 µM AP30663 did not significantly affect the K_ir_3.1/K_ir_3.4 (I_KACh_), K_V_1.5 (I_Kur_), K_V_7.1/KCNE1 (I_Ks_), K_V_4.3/KChiP2 (I_to_), K_ir_2.1 (I_K1_) and Ca_V_2.1 (I_Ca,L_) channels (the latter tested up to 30 µM). A concentration-dependent inhibition of K_V_11.1 by AP30663 was observed, with a calculated IC_50_ of 15 or 4 µM depending on assay type. The difference in potency can likely be explained by the differences in temperature, voltage protocols, and K_V_11.1 isoforms investigated (homomeric K_V_11.1a vs. heteromeric K_V_11.1a/K_V_11.b channels). Minor effects of 10 µM AP30663 on peak I_Na_, but a significant inhibition of the late I_Na_ was observed. Selective inhibition of late I_Na_ is known to suppress ventricular arrhythmias, especially in settings of prolonged ventricular repolarization (Antzelevitch et al., 2004; Wu et al., 2004; Undrovinas and Maltsev, 2008; Antoons et al., 2010; Antzelevitch et al., 2011). Late I_Na_ has been found in atrial cardiomoycytes from patients with and without AF, although only at room temperature and not at physiological temperatures (Poulet et al., 2015). Ranolazine, which inhibits various ion channels in addition to also inhibiting the late I_Na_, has demonstrated anti-AF efficacy in a number of studies (Scirica et al., 2007; Miles et al., 2011). Experimental evidence, however, indicates that ranolazine works against AF only at concentrations that also inhibit I_Kr_ and peak I_Na_ in atria (Burashnikov and Antzelevitch, 2013; Du et al., 2014). Late I_Na_ is reduced at higher heart rates and would not be expected to play a significant role during tachyarrhythmias such as AF (Burashnikov and Antzelevitch, 2013). Whether or not the inhibition of late I_Na_ seen by 10 µM AP30663 contributes to any antiarrhythmic activity of the compound is therefore unknown.

AP30663 consistently led to concentration- and dose-dependent prolongation of atrial refractoriness in *ex vivo* and *in vivo* experiments. Even though K_Ca_2 channels are expressed in both atria and ventricles in guinea pigs (Kirchhoff et al., 2019), targeting K_Ca_2 channel has previously been shown to be atrial selective in a wide range of species, including measurement on human heart tissue (Diness et al., 2010; Qi et al., 2014; Skibsbye et al., 2014; Zhang et al., 2014; Haugaard et al., 2015; Diness et al., 2017). Similarly, in the current study AP30663 was found to predominantly prolong the atrial refractoriness as compared to ventricular repolarization (QTcH). The QTcH prolongation (8 ms as compared to TMC) appeared to reach a plateau already at 1 µM. Considering the *in vitro* IC_50_ of AP30663 on KCa2 (~1 µM) and Kv11.1 (4–15 µM) and the mRNA expression of KCa2 and KV11.1 channels in guinea pig ventricles (Kirchhoff et al., 2019), the QTcH prolongation could be speculated to arise from combined contribution of ventricular KCa2 and Kv11.1 inhibition. The apparent observed plateau seen for QT prolongation could however also indicate that Kv11.1 inhibition has a minor impact. At this stage the exact contribution to the minor QT prolongation from KCa2 and Kv11.1 inhibition can only be speculative. We did not see any effects of AP30663 on QRS-duration, which correlates well with the lack of effects on peak Na_V_1.5 currents stimulated at 1 Hz. In addition to prolongation of AERP, the isolated guinea pig heart AP30663 also slows heart rate and prolongs the PQ-interval, suggesting effects on nodal tissue. This is in line with findings from mouse demonstrating that ablation of K_Ca_2.2 reduces the firing of the AV node(Zhang et al., 2008) and pharmacological inhibition of K_Ca_2 channels by apamin reduces the spontaneous firing of the sinus node(Torrente et al., 2017). Sex specific differences in the importance of SK channels for cardiac ventricular electrophysiology have been reported (Chen et al., 2018). However, we did not observe any differences in the response to AP30663 in male and female guinea pigs.

In the brain K_Ca_2 channels contribute to neuronal action potential after hyperpolarization, and inhibition of the channel is known to disturb motor output from the cerebellum. Consequently, AP30663 was designed to have limited CNS exposure, and infusion of AP30663 to conscious mice confirmed this.

## Conclusion

AP30663 was found to inhibit the K_Ca_2 channel by decreasing the calcium sensitivity of the channel, while being selective over several relevant cardiac ion channels. *Ex vivo* and *in vivo* experiments confirmed the ability of the drug to prolong the AERP in a concentration-dependent fashion with minor effects on the QT interval. Based on this profile, AP30663 is an attractive compound for investigating if K_Ca_2 channel inhibition can be used as a novel antiarrhythmic therapy.

## Limitations

Although the calcium shift in calcium sensitivity was only investigated for K_Ca_2.3 it is likely that the inhibitory mechanism of AP30663 on K_Ca_2.1 and K_Ca_2.2 is similar based on the high sequence homology between the three isoforms. The selectivity of the compound was investigated using overexpression systems and not on native cardiomyocytes. Hence, some of the complexity of native cardiac ion channel complexes, which potentially could impact the selectivity of the compounds, might have been missed. However, the obtained selectivity data on AP30663 combined with our *ex vivo* and *in vivo* ECG data supports that AP30663 does not have major impact on other cardiac ion channels. Moreover, for calcium recordings we only performed 3 experiments, which could limit the conclusion. However from our *ex vivo* and *in vivo* recordings we did not observe signs of calcium channel inhibition (e.g. PR interval prolongations and blood pressure drop).

## Data Availability Statement

All datasets generated for this study are included in the article/**Supplementary Material**.

## Ethics Statement

The animal experiments were performed under the license from the Danish Ministry of Justice (2018-15-0201-01430 & 2016-15-0201-00850) and in accordance with the Danish guidelines for animal experiments according to the European Commission Directive 86/609/EEC.

## Author Contributions

SB, RS-V, LF, LA TS, and KM contributed with acquisition, interpretation and analysis of data. US, MG, and NE contributed with conception and design of the study. BB and JD wrote the first draft of the manuscript and interpreted and analysed the data, and contributed with conception and design of the study. All authors contributed to manuscript revision, read and approved the submitted version.

## Funding

This work was supported by the Innovation Fund Denmark, Wellcome Trust (grant 100406/Z/12/Z), and the European Union’s Horizon 2020 research and innovation programme under the Marie Skłodowska-Curie grant agreement 675351.

## Conflict of Interest

BB, RS-V, TS, NE, US, LA, SB, MG, and GD are employed by and/or have interests in Acesion Pharma and/or are inventors of Acesion Pharma patents within the field of SK channels.

The remaining authors declare that the research was conducted in the absence of any commercial or financial relationships that could be construed as a potential conflict of interest.
