# Supplementary material for: Mechanisms of Action of the KCa2-Negative Modulator AP30663, a Novel Compound in Development for Treatment of Atrial Fibrillation in Man
Source: Front Pharmacol. 2020 May 6;11:610. doi: 10.3389/fphar.2020.00610 (PMC7232560; doi:10.3389/fphar.2020.00610)
Supplement: Supplementary file 1 [file DataSheet_1.docx]

Supplementary Material

Supplemental methods, materials and results to:

**Bentzen et al. Mechanisms of action of the KCa2 negative modulator AP30663, a novel compound in development for treatment of atrial fibrillation in man**

**Table of content**

[1 Supplementary materials and methods 2](#_Toc35945837)

[1.1 *In vitro* electrophysiology 2](#_Toc35945838)

[1.1.1 Automated patch clamp experiments 2](#_Toc35945839)

[1.1.2 K_Ca_2 assay 2](#_Toc35945840)

[1.1.3 K_V_11.1 assay 3](#_Toc35945841)

[1.1.4 Na_V_1.5 assay 3](#_Toc35945842)

[1.1.5 Ca_V_1.2 assay 3](#_Toc35945843)

[1.2 Manual patch-clamp recordings 4](#_Toc35945844)

[1.2.1 K_Ca_2.3 inside out recordings 4](#_Toc35945845)

[1.2.2 K_V_11.1a/1b assay (GLP) 5](#_Toc35945846)

[1.2.3 Late-sodium recordings 5](#_Toc35945847)

[1.3 Two-electrode voltage-clamp 6](#_Toc35945848)

[1.4 Animal experiments 6](#_Toc35945849)

[1.4.1 Isolated perfused guinea pig heart experiments 7](#_Toc35945850)

[1.4.2 Closed chest rat AERP 7](#_Toc35945851)

[2 Supplementary figures 8](#_Toc35945852)

[3 Supplementary tables 9](#_Toc35945853)

[4 Reference list 10](#_Toc35945854)

# Supplementary materials and methods

## *In vitro* electrophysiology

### Automated patch clamp experiments

K_Ca_2.1-3 (SK1-3), K_V_11.1 (hERG), peak Na_V_1.5 and Ca_V_1.2 experiments were done using an automated whole-cell patch-clamp system (QPatch 16 HT) with single-hole Qplates (Sophion, Denmark). The Qpatch automatically generates giga seals, whole-cell formation, compound application, voltage-clamping and recording of current. On the day of experiment, cells were trypsinized with Detachin (Genlantis, CA, USA) and resuspended in Serum Free medium (C5467 SAFC, Buchs, Switzerland) containing 25 mM Hepes, 0.04 mg/ml Soy bean trypsin inhibitor (T6522 Sigma) and 100U/ml penicillin/streptomycin.

### K_Ca_2 assay

Experiments were conducted by automated whole-cell patch-clamping on HEK293 cells stably expressing hK_Ca_2.1, hK_Ca_2.2 or hK_Ca_2.3 (hSK1-3). The cell lines were established as described in^1^. The cell lines were obtained from NeuroSearch A/S (Ballerup, Denmark) and cultured in Dulbecco’s modified Eagle’s medium (DMEM1965, Substrat og sterilcentralen, University of Copenhagen, Denmark) supplemented with 10 % fetal bovine serum (Biowest, France), 100 U/ml of penicillin/streptomycin (Sigma, Germany) and 100 µg/ml geneticin (Gibco, USA).

The recordings were performed in symmetrical K^+^ solutions. The extracellular solution consisted of (in mM): CaCl_2_ 0.1, MgCl_2_ 3, KCl 150, HEPES 10 and glucose 10, pH = 7.4 adjusted with KOH, and the intracellular solution consisted of (in mM): 154 KCl, 10 HEPES, 8.106 CaCl2, 1.167 MgCl2 and 10 mM EGTA. CaCl_2_ is added to give calculated free concentrations of Ca^2+^ of 0.4 µM and MgCl_2_ is added to give a free concentration of 1 mM. Free concentrations were established as previously described^2^. In order to maintain a total [K^+^] of 156 mM (corresponding to the concentration in the extracellular ringer) a fixed amount of KOH (15 mM) was added, thereafter pH was adjusted with HCl to pH = 7.2.

Currents were elicited by a linear voltage ramp from –80 mV to +80 mV (200 ms in duration) applied every 5^th^ second from a holding potential of 0 mV. Recordings were made before and after application of drug. Data were sampled at 10 kHz, four-order Bessel filter, cut-off frequency 3 kHz and 80 % Rs compensation.

The compound application protocol consisted of 9 recording periods lasting from 50-200 s: 1) Baseline recordings in extracellular solution; 2) Application of the positive control 100μM methyl-bicuculline (Sigma), which is characterized by full efficacy, fast on-and off-rate; 3-4) Wash-out; 5-9) Increasing concentrations of AP30663 (in µM: 0.1; 0.3; 1.0; 3.0; 10.0 and 30) to establish an IC_50_-value. Currents were compensated for run-down. Potency was quantified as the concentration needed to inhibit half of the K_Ca_2 channel activity recorded at -80 mV and reported as an IC_50_ value. All drug effects were normalized to the observed full inhibitory effect of methyl-bicuculine.

### K_V_11.1 assay

Experiments were conducted by automated whole-cell patch-clamping on CHO-K1 cells stably expressing hK_V_11.1a cultured in DMEM F12 (Sigma-Aldrich Chemie GmbH, Steinheim, Germany) supplemented with 10 % foetal bovine serum (Biowest, France), 100 U/ml of penicillin/streptomycin (Sigma, Germany), 100 µg/ml geneticin (Gibco, USA), 100µg/mL hygromycin (Sigma-Aldrich) and 2 mM Glutamin (Gibco, Paisley, UK).

Measurements were performed with physiological solutions. The extracellular solution consisted of (in mM): NaCl 145; KCl 4; CaCl_2_ 2; MgCl_2_ 1; 10 HEPES and 10 glucose. pH=7.4 adjusted with NaOH. The intracellular solution contained (in mM): KCl 120; KOH/EGTA 31.25/10; CaCl_2_ 5.4; MgCl_2_ 1.75; HEPES 10; 4 mM Na_2_ATP (pH adjusted with KOH to 7.2).

Cells were voltage clamped at a holding potential of –90 mV, and K_V_11.1 currents were elicited every 7^th^ s by stepping to +20 mV for 2 s and then to –50 mV for 2 s in order to record tail-currents. Data were sampled at 10 kHz, four-order Bessel filter, cut-off frequency 3 kHz and 80 % Rs compensation.

The application protocol consisted of 11 recording periods lasting 70-140 s: 1-2) baseline recordings in extracellular solution.3-7) increasing concentration of AP30663 (1, 3, 10, 30, 100 μM).8-10) wash-out. 11) Dofetilide 500 nM as a positive control. Potency was quantified as the concentration needed to inhibit half of the K_V_11.1 tail current recorded at -50 mV and reported as an IC_50_ value. All drug effects were normalized to the observed full inhibitory effect of dofetilide.

### Na_V_1.5 assay

The effect of AP30663 on Na_V_1.5 peak current was examined using HEK293 cells stably expressing rNa_V_1.5. The HEK293-rNa_V_1.5 (obtained from NeuroSearch A/S (Ballerup, Denmark)) were cultured in DMEM1965, (Substrat og sterilcentralen, University of Copenhagen, Denmark) supplemented with 10 % fetal bovine serum (Biowest, France), 100 U/ml of penicillin/streptomycin (Sigma, Germany).

rNa_V_1.5 currents were recorded with an extracellular solution containing (in mM): 145 NaCl, 2 CaCl2, 1 MgCl2, 10 HEPES, 4 KCl, 10 glucose, pH 7.2, intracellular solution that contained (in mM) 10 NaCl, 135 CsF, 5/1 CsOH/EGTA, 10 HEPES, 4 Na-ATP, pH 7.3 (adjusted with CsOH).

rNa_V_1.5 currents were elicited every 1000 milliseconds by a 50 ms depolarizing step to -20 mV from a holding potential of -120 mV (a total of 40 pulses). Data were sampled at 25 kHz, eighth-order Bessel filter, cutoff frequency 3 kHz.

The application protocol consisted of 3 recording periods (100 s duration). 1) baseline. 2) AP30663 10 µM. 3) 10 µM Tetracaine (positive control). The peak current at the 40th pulse was found and % change from baseline was found as: %-inhibition= input-Baseline/Full response-Baseline.

### Ca_V_1.2 assay

Effect of AP30663 on L-type calcium channels was investigated by automated patch clamping (Qpatch, Sophion) using a hCa_V_ 1.2 stable cell line (Alpha1C, Beta2C and Alpha2delta) (SB Drug Discovery).

Ca_V_1.2 currents were recorded with an extracellular solution containing (in mM): 143 mM TEA-Cl, 1 mM MgCl2, 5 mM BaCl2 and 10 mM HEPES and 15 mM Glucose (pH 7.4 adjusted with NaOH). The intracellular solution contained (in mM) 27 mM CsF, 112 mM CsCl, 2 mM NaCl, 8.2 mM EGTA, 10 mM HEPES and 4 mM MgATP; the pH was adjusted to 7.25 with CsOH.

Cells were voltage clamped at -120 mV and CaV1.2 current were elicited every 5^th^ s by a 300 ms test pulse at 0mV, and then returned back to -120 mV.

The application protocol consisted of 10 recording periods (50 s duration): 1-2) baseline. 3-7) increasing concentrations of AP30663 (1, 3, 10, 30 (in μM)), followed by the positive control, Nifedipine (10μM).

Potency was quantified as the concentration needed to inhibit half of the Ca_V_1.2 peak current recorded at 0 mV and reported as an IC_50_ value. All drug effects were normalized to the observed full inhibitory effect of Nifedipine.

## Manual patch-clamp recordings

### K_Ca_2.3 inside out recordings

HEK293 cells stably expressing hK_Ca_2.3 were used for inside out recordings. Cells were cultured as described for the automated K_Ca_2 assay. Recordings were done in symmetrical K^+^ solutions in similar solutions as described for the automated K_Ca_2 assay. However, intracellular solutions containing 9 different free Ca^2+^ concentrations (0.01 - 30 µM) were used to study the effect of AP30663 on the calcium activation of the channel. The composition of these intracellular solutions was determined as described in ^2^.

Inside out voltage-clamp recordings were performed using a HEKA EPC9 amplifier and the Patchmaster software (HEKA Elektronik, Germany) at room temperature. Patch pipettes were pulled using a horizontal DMZ Universal Puller (Zeitz, Germany) with resistances of 2 MΩ when filled with intracellular solution. Cells were plated on coverslips pre-treated overnight at 37ºC with 50 mg/ml poly-L-lysine (Sigma, Germany) to get firmer cell attachment. K_Ca_2 currents were elicited by a voltage ramp protocol ranging from -80 mV to +80 mV from a holding potential of 0 mV. The current at -80 mV was used for the analysis. Only currents from the last 10 data points of every application corresponding to the steady state were considered for EC_50_ calculations.

To calculate the EC_50_ of calcium activation of the channel the individual recordings were normalized to the lowest calcium concentration (0.01 µM) for total inactivation and highest calcium concentration (30 µM) for maximum activation of the channel. Individual EC_50_ values for each experiment were calculated using the equation:

$$Y=Ymin+\frac{(Ymax-Ymin)}{1+{10}^{(logEC50-X)\times HillSlope}}$$

where X is the log of dose of calcium and Y is the normalized measured current with variable Hill slope.

### K_V_11.1a/1b assay (GLP)

The study was performed at Centre de Recherches Biologiques (CERB), France, in compliance with the guidelines concerning Good Laboratory Practice (GLP).

HEK-293 cells stably transfected with hERG-1a/1b cDNA were obtained under a Biomaterials license withWARF0s consent from Dr. January at the University of Wisconsin-Madison, USA. The cells were maintained in culture using (DMEM) supplemented with foetal bovine serum (10%), non-essential aminoacids (1%) and geneticin (0.4 mg/mL).

Cells were voltage clamped using a AXOPATCH 200B (AXON instrument) to -80 mV, depolarised to 0 mV for 5 sec allowing activation of K_V_11.1a/1b current and repolarised to -50 mV for 5 sec allowing hKV11.1a/1b tail current to deactivate. This experimental procedure was repeated at a frequency of 0.06 Hz. Currents were filtered at 1 kHz and acquired at the frequency of 2 kHz. Amplitude of K_V_11.1 tail current was measured during the repolarizing pulse from 0 to -50 mV. The intracellular solution contained in mM: KCl 130, MgCl_2_ 1, HEPES 10, ATPMg 5, EGTA 5, pH adjusted to 7.2 with KOH. Cells were perfused with Tyrodes solution (mM): NaCl 137, KCl 4, MgCl_2_ 1, CaCl_2_ 1.8, Glucose 10, HEPES 10, pH adjusted to 7.4 with NaOH), the vehicle (DMSO) and subsequently with Tyrodes solution containing AP30663 for 5 minutes until steady state was reached for each perfusion period. Three ascending concentrations of AP30663 were tested (1, 10 and 100 µM). All solutions were heated to 35.5 ± 0.5 C^o^. Based on the inhibition of tail current by AP30663 at these three concentrations an IC_50_ value was established.

### Late-sodium recordings

Electrophysiological recordings of late-sodium currents were performed in HEK-293 cells transiently transfected with hNa_V_1.5 (1 µg DNA) and eGFP (0.2 µg DNA) with lipofectamine (Invitrogen, USA). The cells were trypisinized 24± 2 hours after transfection and allowed to attach on coverslip for an hour before patching commenced. Whole-cell currents were acquired at room temperature with an EPC-9 amplifier data acquisition system with Patchmaster software (HEKA Elecktronik, Germany). Borosilicate glass pipettes were pulled using a puller (Sutter instrument, CA, USA) to attain a tip resistance of 1.5-3.5 MΩ when filled with the intracellular solution. The extracellular solution consisted of (in mM): 130 NaCl, 5 CsCl, 2 CaCl_2_, 1.2 MgCl_2_, 10 HEPES and 5 glucose; pH adjusted to 7.4 with CsOH, and the intracellular consisted of (in mM): 60 CsCl, 70 aspartic acid, 70 CsOH, 1 CaCl_2_, 1 MgCl_2_, 11 EGTA, 10 HEPES, 5 Na_2_-ATP; pH adjusted to 7.2 with CsOH. Late sodium (late I_Na_) currents were measured by patch clamp technique using whole-cell configuration. Cells were voltage-clamped at -100 mV and depolarized from -100 mV to -20 mV for 300 ms every 5^th^ second. ATX II (5 nM) was applied after Na_V_1.5 peak current stabilization for the augmentation of late sodium current and Tetrodotoxin (TTX 10 µM) was used at the end of the recording to inhibit the sodium current. Experiments with unstable leak current, series resistance or experiments where less than 50 % of the current could be inhibited by TTX (10 µM) at the end of the recording were excluded.

The application protocol consisted of 4 recording periods: 1) baseline, until peak current stabilized. 2) ATXII 5 nM to augment late-sodium current. 3) ATXII 5 nM+AP30663 10 µM. 4) ATXII 5 nM + TTX 10 µM.

Late INa was defined as the current amplitude recorded 200 ms after the initial membrane depolarization to -20 mV (300 ms duration) from a holding potential of -100 mV. Because the effect of ATX on late-sodium current never saturates we evaluated the effect of AP30663 by comparing the slope of the late-INa current vs time plot before and after co-application of AP30663. The slope was found by linear regression on the last 10 points of each liquid period.

## Two-electrode voltage-clamp

*Xenopus laevis* oocytes were purchased from EcoCyte Bioscience (Castrop-Rauxel, Germany). cRNA for injection was prepared from linearized plasmids using the mMESSAGE mMACHINE T7 kit (Ambion, TX, USA) according to the manufactories’ protocol. RNA concentrations and quality were assessed by UV spectroscopy (NanoDrop, Thermo Scientific, Wilmington, USA) and gel electrophoresis. The following concentrations of cRNA were used (µg/µL): K_V_7.1+KCNE1 0.08 + 0.02; K_ir_2.1 0.4; K_V_4.3+ KChIP2 0.01+0.01; K_ir_3.1+K_ir_3.4 0.04+0.04; K_V_1.5 0,1. 50 nL cRNA was injected and the two-electrode voltage clamp recordings were performed after 2-3 days of incubation (19^o^C) using a Dagan CA-1B amplifier. Borosilicate glass recording electrodes Module Ohm, Herlev, Denmark) were pulled on a DMZ-Universal Puller (Zeitz Instruments, Martinsried, Germany)and had a resistance of 0.5–1 MΩ when filled with 2 M KCl. Oocytes were superfused with Kulori solution (in mM: NaCl 90, KCl 4, MgCl_2_ 1, CaCl_2_ 1, 4-(2-hydroxyethyl)-1-piperazineethanesulfonic acid 5, pH=7.4 with NaOH, room temperature).

The compound application protocol consisted of 3 recording periods each lasting 5 min: 1) baseline. 2) AP30663 10 µM. 3) positive control (K_V_7.1/KCNE1: JNJ303 1 µM; K_ir_2.1: Ba^2+^100 µM; K_V_4.3+ KChIP2: 4-AP 4 mM; K_ir_3.1+K_ir_3.4: Tertiapine-Q 1 µM; K_V_1.5: 4-AP 4 mM).

The following voltage protocols were used to elicit the K^+^ currents (holding membrane potential -80mV):

K_V_7.1/KCNE1: currents were elicited by clamping the membrane potential to +20 mV for 4 s followed by a 1 s step to -40 mV. Steady state current measured at +20 mV were used for the analysis.

K_V_1.5 and K_V_4.3/KChIP2: Currents were evoked by changing the membrane potential to +20 mV or 0 mV for 2 s respectively. Steady state current amplitudes of _K_V1.5 and peak current amplitudes of K_V_4.3/KChIP2 were used for analysis.

K_ir_2.1 and K_ir_3.1+K_ir_3.4: Currents were elicited by ramp protocol from -100 mV to +80 mV (5 s duration). The peak current measured at -120 mV was used to assess the effect of AP30663

Data acquisition was performed with the Pulse software (HEKA Elektronik, Lambrecht/Pfalz, Germany). Data are represented as %-change from baseline.

## Animal experiments

The mice and rats were housed in groups of 4-6 or 2-4 in high-top cages with wood shavings as bedding, whereas guinea pigs were housed in groups of up to 20 with straw bedding under constant climatic conditions (22°C) at the Department of Experimental Medicine, University of Copenhagen. The animals had access to clean water and standard laboratory diet ad libitum and were kept at a 12 hour light-dark cycle.

### Isolated perfused guinea pig heart experiments

A total of 12 female Guinea pigs from the Dunkin Hartley strain (HsdPoc:DH)( Charles River, Scanbur A/S, Karlslunde, Denmark) weighing between 350 and 600 g were used; 6 were time matched controls, and 6 received AP30663. The isolated heart was prepared as follows: After anesthetizing with 200 mg/kg pentobarbital and lidocaine hydrochloride 0.150 ml/100 g body weight i.p., (Glostrup Apotek, Denmark), a dose of 1000 IU/kg heparin was injected intravenously. Respiration was maintained by artificial ventilation through a cannula in the trachea (volume, 12 ml/kg; rate, 60 strokes/min). Upon thoracotomy, a perfusion cannula was inserted and fixed in the aorta for retrograde perfusion. Hearts were mounted in a vertical Langendorff set-up (Hugo Sachs Elektronik, Harvard Apparatus GmbH, Germany) and perfused with Krebs-Henseleit solution( in mM L^-1^: NaCl 120.0, NaHCO_3_ 25.0, KCl 4.0, MgSO_4_ 0.6, NaH_2_PO_4_ 0.6, CaCl_2_ 2.5, Glucose 11.0, saturated with 95% O_2_ and 5% CO_2_, 37°C, pH 7.4) at a constant perfusion pressure of 60 mmHg). The electrocardiogram (ECG) was obtained with three ECG electrodes placed in close proximity to the heart. A pacing electrode was placed on the right atrium to stimulate the atria and measure atrial refractory periods (AERP). All data were acquired at 2 KHz using the 16-channel PowerLab system (ADInstruments, Oxford, UK), and monitoried by LabChart 7 software (ADInstruments).

Baseline recordings with no compound present were made for at least 20 minutes and continued until the ECG morphology and AERP recording were stable for at least 10 minutes before adding test compound. After the baseline recording, three 20-minute episodes followed in which the heart was perfused with increasing concentrations of AP30663 (1 µM, 3 µM, and 10 µM). Time matched control hearts underwent the same procedure except no test compound was added at any time to these hearts. During the entire experiment the ECG was monitored and recorded and every five minutes the AERP was measured by applying electrical stimulation (4 times rheobase) with a fixed interval of 200 ms (S1S1 stimulation) and for every 10^th^ beat an extra stimulus (S2 stimulation) was applied with 1 ms increments. The AERP was defined as the longest S1-S2 interval failing to elicit an action potential. Between the AERP recordings, the heart remained unpaced. QT-intervals were analysed at intrinsic heart rates.

### Closed chest rat AERP

A total of 7 male Sprague Dawley rats (Janvier Labs, Le Genest-Saint-Isle, France) weighing 300-600 g were used for the closed chest preparations. The rats were randomized to either the AP30663 group (n=3) or the time matched control (TMC) group (n=4) that received the same volume of vehicle as the AP30663 group.

The rats were anaesthetized with 3 % isofluran/oxygen and an intravenous catheter, used for drug infusion, was placed in the femoral vein. The intra-cardiac catheter (Millar Inc., US) was placed in the right atrium through the jugular vein which allowed in order to pace the heart and determine AERP. Needle ECG electrodes were placed in each limb to record the ECG. During the entire experiment the ECG was monitored. The temperature of the rats was monitored and kept stable at 37^o^C throughout the experiment with a heating lamp. The AERP was measured by applying electrical stimulation via 2 of the 8 electrodes on the intra-cardiac catheter (5 times rheobase) with a fixed interval of 120 ms (S1 stimulation) and for every 10^th^ beat an extra stimulus (S2 stimulation) was applied with 1 ms increments. The AERP was defined as described for the isolated heart experiments. The heart remained unpaced between the AERP recordings. Baseline AERP recordings were made every 5^th^ minute for 20 minutes before adding AP30663. Hereafter, two 20-minute episodes followed in which the animal was injected with increasing doses of AP30663 (5 mg/kg and 10 mg/kg) or equivalent volumes of vehicle for the time matched control animals. AERP was measured 0.5, 2, 4, 6, 10 and 15 minutes after each injection.

# Supplementary figures


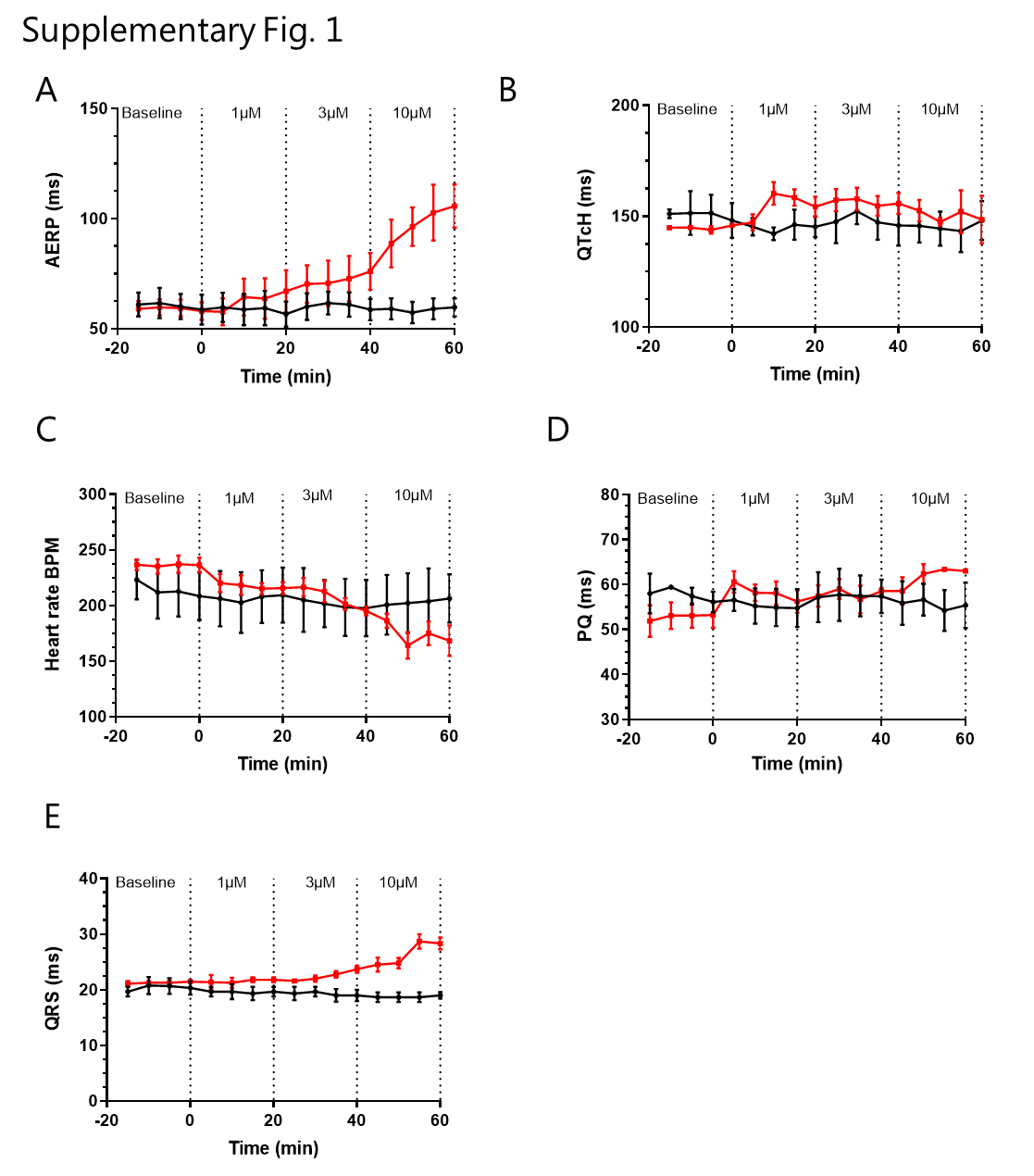


**Supplementary figure 1. AP30663 prolongs the atrial refractory period in isolated perfused male guinea pig hearts.** Graph demonstrating the effect of increasing concentrations of AP30663 on AERP (A), QTcH-interval (B), Heart rate (C), PQ-interval (D) and QRS-duration (E) as compared to time matched control experiments (TMC) (AP30663: n=3; TMC: n=3).

# Supplementary tables

| AP30663 concentration |  | 1µM | 3µM | 10µM |
| --- | --- | --- | --- | --- |
| AERP (ms) | TMC | -2±1 | 0±3 | 1±5 |
|  | AP30663 | 9±6 | 18±4 | 48±7 |
|  | p | 0.125 | 0.055 | 0.016 |
| QTcH (ms) | TMC | -3±2 | -2±7 | 0±2 |
|  | AP30663 | 8±5 | 10±5 | 3±11 |
|  | p | 0.302 | 0.421 | 0.815 |
| HR (BPM) | TMC | 1±5 | -11±9 | -2±11 |
|  | AP30663 | -21±2 | -41±5 | -68±8 |
|  | p | 0.032 | 0.043 | 0.025 |
| QRS (ms) | TMC | -1±0 | -1±0 | -1±1 |
|  | AP30663 | 0±0 | 2±0 | 7±1 |
|  | p | 0.101 | 0.004 | 0.004 |
| PQ (ms) | TMC | -1±2 | 1±1 | -1±3 |
|  | AP30663 | 3±0 | 5±3 | 8±1 |
|  | P | 0.181 | 0.239 | 0.130 |

**Supplementary table 1**: Effect of AP30663 on isolated perfused male guinea pig hearts. Changes in AERP, QTcH, HR, PQ and QRS (Δdrug – baseline) for each group (TMC and AP30663) calculated at the end of drug period (20 min, 40 min and 60 min). P-values refer to the comparison of Δ-values between TMC and AP30663 groups.

# Reference list

1. Strøbæk D, Teuber L, Jørgensen TD, Ahring PK, Kjær K, Hansen RS, Olesen SP, Christophersen P, Skaaning-Jensen B. Activation of human IK and SK Ca 2+-activated K + channels by NS309 (6,7-dichloro-1H-indole-2,3-dione 3-oxime). *Biochim Biophys Acta - Biomembr*. 2004;1665:1–5.

2. Strøbæk D, Hougaard C, Johansen TH, Sørensen US, Nielsen EO, Nielsen KS, Taylor RD, Pedarzani P, Christophersen P. Inhibitory gating modulation of small conductance Ca2+-activated K+ channels by the synthetic compound (R)-N-(benzimidazol-2-yl)-1,2,3,4-tetrahydro-1-naphtylamine (NS8593) reduces afterhyperpolarizing current in hippocampal CA1 neurons. *Mol Pharmacol*. 2006;70:1771–1782.
